# The incidence and factors of hip fractures and subsequent morbidity in Taiwan: An 11-year population-based cohort study

**DOI:** 10.1371/journal.pone.0192388

**Published:** 2018-02-15

**Authors:** Kai-Biao Lin, Nan-Ping Yang, Yi-Hui Lee, Chien-Lung Chan, Chi-Hsu Wu, Hou-Chuan Chen, Nien-Tzu Chang

**Affiliations:** 1 School of Computer & Information Engineering, Xiamen University of Technology, Xiamen, China; 2 Department of Information Management, Yuan Ze University, Taoyuan, Taiwan; 3 Department of Surgery & Orthopedics, Keelung Hospital, Ministry of Health & Welfare, Keelung, Taiwan; 4 Institute of Public Health and Community Medicine Research Center, National Yang-Ming University, Taipei, Taiwan; 5 School of Nursing, College of Medicine, National Taiwan University, Taipei, Taiwan; 6 Department of Nursing, School of Nursing, College of Medicine, Chang-Gang University, Taoyuan, Taiwan; 7 Innovation Center for Big Data and Digital Convergence, Yuan Ze University, Taoyuan, Taiwan; 8 Department of Bioengineering, University of Strathclyde, Glasgow, United Kingdom; 9 Department of Orthopedics, Taoyuan Hospital, Ministry of Health & Welfare, Taoyuan, Taiwan; Garvan Institute of Medical Research, AUSTRALIA

## Abstract

Hip fractures are a major problem to elder population, but subsequent morbidity is unclear about environmental factors and socioeconomic conditions. The study aims to investigate the incidence of hip fractures treated by the surgery; to compare the sequelae and temporal trends of hip fractures; to evaluate the seasonal effects in the subsequent short-term and long-term morbidities after hip fractures. A cohort study design is conducted using national health research datasets between 2000 and 2010. The ICD-9-CM diagnostic codes were utilized to investigate the incidence of hip fractures and the corresponding treatments. Hierarchical modeling was used to analyze the factors associated with various types of hip fractures. The results indicated that females had a lower incidence in the 30–44 age group, but a significantly higher incidence than males among those aged 60 years or older (adjusted rates 232.1 vs. 100.3 per 100,000 persons, p<0.001). The incidence of hip fractures in the low-income group showed no significant difference compared to that in the general population. There was a temporal trend of a 8.6% increase in the incidence of all types of hip fractures over the period of 2000–2010. A summer-winter variation is observed among the elderly. Hip fractures and subsequent morbidity are increasing in Taiwan’s aging society. Older age, female gender, and time periods were independent risk factors for subsequent morbidities after surgical treatment. The result of this study is useful to the healthcare policy makers and to raise the public awareness of hip fractures.

## Introduction

Hip fractures are a major threat to the survival of affected individuals associated with a high mortality rate (14–36%) and morbidity [1, 2]. Fatalities are often not caused by the hip fracture itself, but associated traumatic injuries [2]. It has been estimated that the mortality of the hip fracture patients in thirty days is 19%. The mortality in one year after hip fracture surgery is 20% to 30% [3]. After hip-fracture surgery, only few patients regained their physical function [4, 5]. Understanding traumatic injury and its subsequent adverse impacts are preventive issues and public concerns.

Several risk factors associated to the hip fractures have been identified. Among these factors, the age is the most important one. A recent systematic review concluded that hip fractures are more likely to be found in older individuals [6]. Some geographic variation in the incidence of hip fractures among different regions has been documented in the last few decades. To the best of our knowledge, the rate of hip fractures might be lower in Asian but it is increasing [7]. This variation in the distribution of hip fractures in different regions of the world shows that etiological and environmental factors such as a north-south gradient seen in European studies play a role in hip fractures [8]. It is important to explore the population-based data about hip fractures and to identify related risk factors in Asia and worldwide. Patients with hip fractures may need various clinical interventions and multi-disciplinary rehabilitation for up to two years to enhance their physical function. Shyu et al. (2016) collected systematic review papers and reported that health outcomes could be significantly improved after hip fractures by interdisciplinary care [9]. Prognostic factors for the outcome after hip fractures are important, but can be obtained only by analyzing data from hospital stays.

The ageing of the population has raised issues in Asia. Hip fractures are a serious geriatric issue in Taiwan [10], and lead to more medical demands and expenditure. There are few studies about hip fractures in Asia, except from Japan [11, 12]. Therefore, there is an urgent need to initiate epidemiological studies in Asia to assess potential changes in risk factors for hip fractures in order to allow health administrators to plan policies for future care [12]. The majority of previous studies have focused only on the short-term consequences [7, 13]. There is still little knowledge about long-term health outcomes and the secondary prevention after hip fractures, particularly among the total population of hip fractures in Asian countries [7].

### Aims

The aims of this study were: (1) to investigate the cumulative incidence and hospital course of Taiwanese with hip fractures, (2) to compare the morbidity and temporal trends of patients with different types of hip fractures, and (3) to evaluate the seasonal effects in the incidence of various short-term and long-term morbidities in Taiwanese patients with hip fractures.

## Methods

### Study subjects

This longitudinal cohort study, an 11-year retrospective cohort is based on the National Health Insurance (NHI) research datasets were randomly sampled from the entire population in Taiwan. Every claimant in the NHI Program was included in the national population. With the use of a random number generator, one million beneficiaries were selected from NHI research datasets. The datasets related to this million cohort used in this study were from 1 January 2000 to 31 December 2010. The inpatient expenditures by admission and prescription datasets which include treatment codes were used for analysis. A total of 5084 patients who had received surgical treatment for hip fractures during the study period were identified by diagnosis and treatment codes. The subsequent morbidity was examined by diagnostic codes.

### Data validity, reliability and ethical considerations

This study is based on nationwide data obtained from the NHI, universal national health insurance program, which covers 99.62% of the total population of Taiwan and has been consistently validated. Therefore, the rate of loss follow-up won’t affect or bias the result significantly [14]. To ensure the protection of patients’ personal information, all datasets were passed through a scrambling procedure before being analyzed. The overall demographic distribution in this database is population-based, nationwide, and representative [15].

This study was approved by the research ethics committee. The permission to analyze data was granted on March 2, 2014. This study was a secondary database (National Health Insurance Research Data (NHIRD)) analysis and the protocol was evaluated by the government research institute, National Health Research Institutes (NHRI), which gave their permission for this analysis (Agreement Number: NHIRD-103-093). The data protection and permission protocols were also approved (Approval Number: TYGH103006) by the Institutional Review Board (IRB) of Taoyuan General Hospital, Ministry of Health & Welfare, which has been certificated by the Ministry of Health and Welfare, Taiwan.

### Definition of variables

The study subjects needed to have matching codes for hip fracture diagnosis and treatment. The diagnostic codes for hip fractures were those in the 820.0–820.1 series (trans-cervical femur fracture), 820.2–820.3 series (per-trochanteric femur fracture), and 820.8–820.9 series (unspecified part of femoral neck fracture), and the treatment codes for hip fractures were joint replacement and open reduction of a femoral neck or shaft fracture. In order to investigate the relationship with subsequent morbidity or associated injuries, nine groups of the following diagnosis codes were evaluated when the morbidity following hip surgery: cerebrovascular accident (ICD-9-CM codes: 430–438), ischemia heart diseases (410–414), pneumonia (480–486), upper gastrointestinal bleeding (578.9), peptic ulcer disease (531–534), renal insufficiency (580–589), new fracture of the trunk (805–809), upper limb fracture (810–819), and lower limb fracture (820–829).

### Statistical analysis

Descriptive statistics including the baseline characteristics in this study were numbers, percentages, crude annual incidence (*Inc*.), which was calculated by dividing the total number of hip fractures with surgery in that specific age- and sex- group by the total number of person years in that group. As differences in crude rates may be possible from the different age distribution in the distinct countries, age-standardized adjusted rates (AR) and their 95% confidence interval (CI) were estimated by the directly standardization method using the standard age-structure of the world population in 2000, the formulae as follows [16, 17]:

AR.±1.96×Inc.×(1−Inc.)×Worldpop.iTWpop.i (1)

Where *i* = age stratum

We also estimated the change of temporal trends with equal weights over the entire time series to the average annual percentage changes (AAPCs) by the Joinpoint Regression Program Version 4.3.1.0 [18, 19]. Potential seasonal variation was compared using the chi-square test by the Statistical Package for Social Sciences for Windows (SPSS for Windows Version 22.0) and the level of significance was set at p = .05.

Multilevel analysis with hierarchical logistic modeling (HLM 7.0 software) [20], which allowed to evaluate the group-level and individual-level factors was used to investigate the factors associated with various types of hip fractures, the individual effects (i.e. gender, socio-economic status (SES), Charleston Co-morbidity Index (CCI), age stratum) and the group effect (period and region). A control group was selected from subjects without hip-fractures from 2000–2010 by one-to-four pair matching for gender, age (within 5 years) and Charleston Co-morbidity Index (CCI). The hypotheses and formulae were:

Level 1 HLM Model

$$Y_{ij}=\beta_{0}+\beta_{1}\times \left(gender\right)+\beta_{2}\times \left(age\;group\;45-59.9\right)+\beta_{3}\times \left(age\;group\;60-74.9\right)+\beta_{4}\times \left(age\;group\;75\;or\;above\right)+\beta_{5}\times \left(SES\right)+\gamma$$ (2)

Level 2 HLM Model

$$\beta_{0}=\gamma_{00}+\gamma_{01}\times (rural\;region)+\gamma_{02}\times (recent\;5yr\;period)+\mu$$ (3)

## Results

### Incidence and characteristics of patients treated for hip fractures in Taiwan

The results indicated that the crude annual incidence (*Inc*.) of hip fractures undergoing surgical treatment ranged from 3.48 to 13.51 per 1,000,000 inhabitants in Taiwan, and the adjusted standard rate (*AR*) was 5.01 to 11.70 per million persons. Females had a lower incidence in the 30–59 age group (1.07 to 1.45 per million persons), but a significantly higher incidence than males among those aged 60 years or older (23.21 vs. 10.03, p<0.001). Between 2000 and 2010, the incidence of hip fractures in the elderly was about two times higher in women than in men. The age-specific incidence rates for hip fractures significantly increased in both genders. The average annual increase rates were 9.5% and 7.6% in males and females respectively. These trends in the incidence of hip fractures monotonically increase over time (Table 1).

**Table 1:** The trends in the incidence of hip fractures undergoing surgical treatment stratified by gender and age stratum.

	*Crude Annual Incidence*	Adj*usted Rates*, AR*	*(95% CI)*		Young age	(30–59.9 y/o)			Old age	(≥60	y/o or above)
Year				Male		Female		Male		Female	
				AR*	*(95% CI)*	AR*	*(95% CI)*	AR*	*(95% CI)*	AR*	*(95% CI)*
2000	3.5	5.0	(4.5, 5.5)	1.4	(1.1, 1.7)	1.1	(0.8, 1.3)	10.0	(9.4, 10.6)	23.2	(22.3, 24.1)
2001	4.7	6.7	(6.2, 7.2)	1.6	(1.3, 1.9)	1.1	(0.9, 1.3)	16.8	(16.1, 17.6)	29.5	(28.5, 30.4)
2002	5.1	6.8	(6.3, 7.3)	2.1	(1.7, 2.4)	1.4	(1.1, 1.7)	13.6	(13.0, 14.3)	31.4	(30.5, 32.3)
2003	6.3	8.0	(7.5, 8.5)	2.3	(1.9, 2.6)	1.3	(1.1, 1.5)	19.3	(18.6, 20.0)	35.5	(34.6, 36.4)
2004	7.5	9.0	(8.5, 9.6)	2.2	(1.9, 2.5)	1.4	(1.2, 1.7)	23.5	(22.8, 24.2)	39.7	(38.8, 40.6)
2005	8.8	9.9	(9.4, 10.5)	2.6	(2.2, 3.0)	1.6	(1.3, 1.8)	23.1	(22.5, 23.8)	45.9	(45.0, 46.8)
2006	8.9	9.6	(9.1, 10.1)	2.2	(1.9, 2.5)	1.6	(1.3, 1.6)	24.6	(24.0, 25.3)	42.7	(41.9, 43.6)
2007	10.5	10.8	(10.2, 11.3)	2.2	(1.8, 2.5)	1.5	(1.3, 1.5)	26.1	(25.5, 26.8)	50.9	(50.0, 51.8)
2008	11.8	11.4	(10.9, 12.0)	2.9	(2.5, 3.2)	1.5	(1.3, 1.5)	27.7	(27.1, 28.4)	52.8	(51.9, 53.7)
2009	13.0	11.9	(11.3, 12.4)	2.6	(2.3, 2.9)	1.5	(1.2, 1.5)	30.2	(29.5, 30.8)	54.7	(53.83, 55.5)
2010	13.5	11.7	(11.2, 12.2)	2.9	(2.6, 3.3)	1.5	(1.2, 1.5)	33.2	(32.6, 33.9)	49.6	(48.86, 50.4)
AAPC** (p-value)	15%	8.6%	(p<0.001)	7.5%	(p<0.001)	3.1%	(p<0.001)	10.6%	(p<0.001)	8.2%	(p<0.001)

***Inc*.:** *Crude Annual Incidence*; *AR: Adj*usted Rates* by standardized age structure in a world population (pop.) unit: per 1,000,000 persons

AAPC**: average annual percentage changes; 95% *CI*: 95% confidence intervals

**Fig 1:**
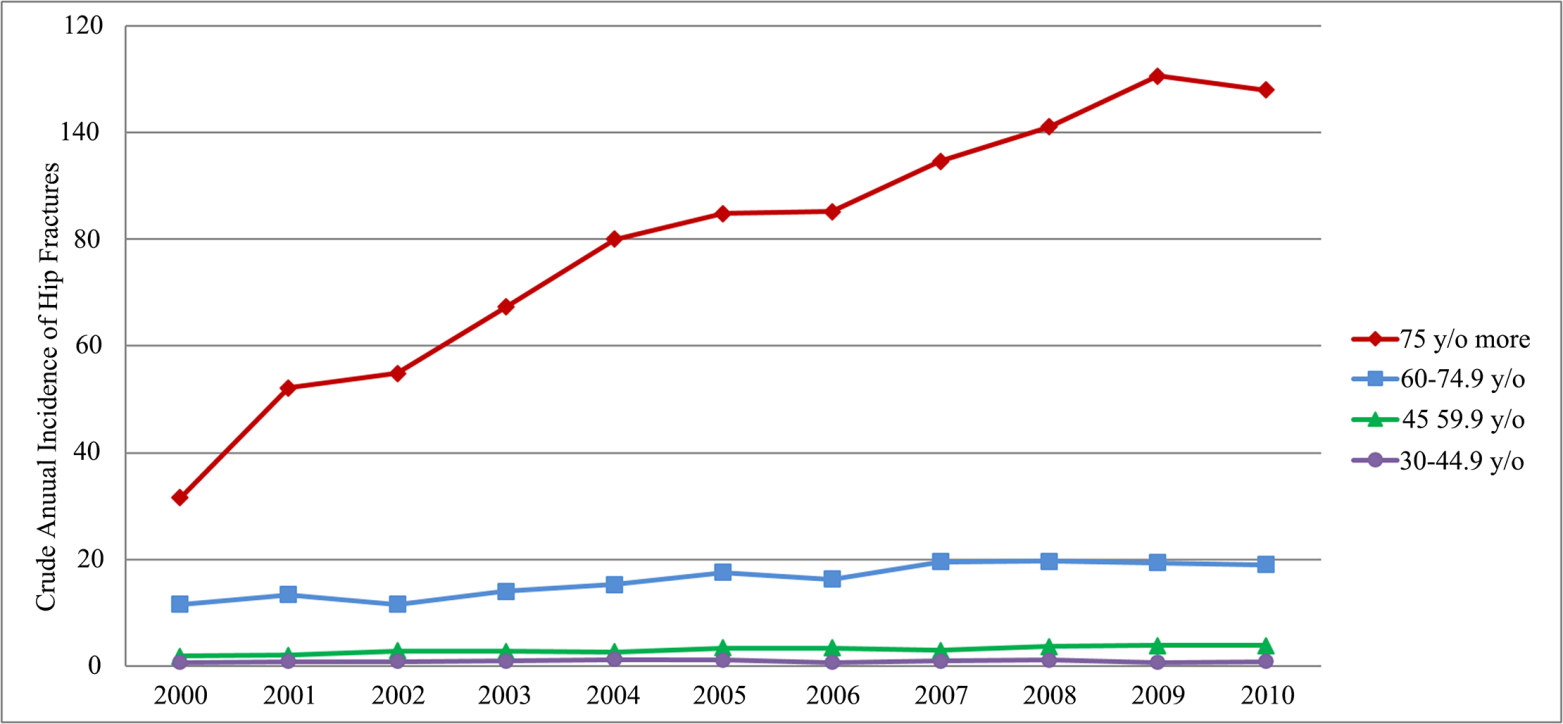
The effects of age and time period on the incidence of hip fractures in Taiwan, 2000–2010.

Fig 1 shows the effect of age across the time on the incidence of hip fractures among the enrolled in-patients. According to this epidemiologic curve and Table 1, the adjusted incidence rate (*AR*.) rose to 100 per 100,000 since 2005. Annual incidence (*Inc*.) increased during the period 2000–2010. The majority of hip fractures occurred in the oldest age stratum of 75 and above, followed by those 60–74, and the incidence rates for those younger than 45 were the lowest. The increasing trend is also most significant in the oldest age stratum of 75 and above.

### Distributions of types of hip fractures and operative methods

**Fig 2:**
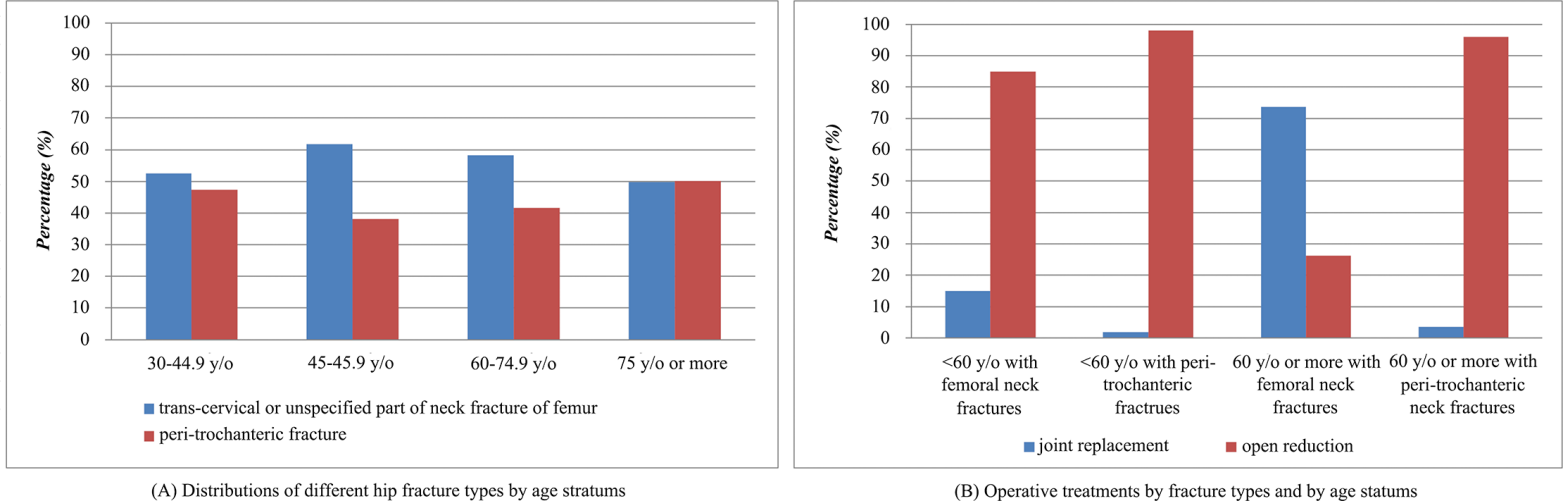
The distributions of different types of hip fractures in the period 2000 to 2010.

According to the ICD-9-CM diagnostic coding principles, peri-trochanteric fractures are coded as 820.2 or 820.3. Trans-cervical fractures or fractures of an unspecified part of the femur neck are coded as 820.0, 820.1, 820.8, or 820.9. They should be treated by either joint replacement or open reduction. Fig 2 shows that the distributions of two types of hip fractures were similar across the age groups in the period 2000 to 2010. Most of the trochanteric fractures received the same treatment which isthe open reduction fractures surgery. Fig 2B also shows, different operative treatment approaches were used in young adults and the elderly when their fracture types were the same. There was an obvious difference in the treatment used in different age-stratums for different types of fractures: there were fewer joint replacements for those below 60 years of age than for those older than 60.

### Hierarchical modeling of predictors of different types of hip fracture

Table 2 shows the adjusted risk factors related to various types of hip fractures by hierarchical logistic modeling. Females had a higher risk of all types of hip fractures than did males. The oldest age stratum (aged 75 or more) has a significantly higher risk. Low-income populations were more likely to live in suburban and rural areas; therefore, their incidence of hip fractures was tested by hierarchical modeling adjusted for regional differences. No significant difference was found between low income population and other populations in this study. This study also found thatin the most recent 5-year period, there was a significant increase of 17% in the incidence of hip fractures compared with the period of 2000–2004.

**Table 2:** Hierarchical linear modeling* of risk factors related to various types of hip fractures.

Risk factors	All hip fracture cases	Femoral neck fractured cases	Per-trochanteric fractured cases
	(n = 5164)	(n = 2781)	(n = 2402)
	AOR (95% CI)	AOR (95% CI)	AOR (95% CI)
**Gender**^**1**^			
Male (n = 2019)	1.00	1.00	1.00
Female (n = 3145)	**1.08 (1.07, 1.09)**	**1.06 (1.05, 1.09)**	**1.01 (1.01, 1.02)**
**Age Stratum**^**1**^			
30–44.9 y/o (n1 = 241)	1.00	1.00	1.00
45–59.9 y/o (n2 = 628)	1.02 (0.99, 1.04)	**1.03 (1.05, 1.12)**	0.99 (0.97, 1.01)
60–74.9 y/o (n3 = 1515)	1.02 (0.98, 1.03)	**1.02 (1.00, 1.04)**	0.99 (0.97, 1.01)
75 y/o or more (n4 = 2780)	1.03 (0.99, 1.06)	1.02 (0.99, 1.04)	**1.02 (1.00, 1.04)**
**Socio-economic level**^**1**^			
Normal population (n = 4950)	1.00	1.00	1.00
Low-income population(n = 214)	1.01 (0.98, 1.02)	1.01 (0.98, 1.04)	0.99 (0.98, 1.01)
**Urbanlization**^**2**^			
Urban	1.00	1.00	1.00
Rural	1.01 (0.96, 1.21)	1.01 (0.79, 1.05)	1.01 (0.99, 1.02)
**Period**^**2**^			
2000–2004	1.00	1.00	1.00
2005–2010	**1.17 (1.16, 1.18)**	**1.10 (1.09, 1.10)**	**1.09 (1.08, 1.09)**

* The control group was selected from the freely hip-fractured subjects by one-to-four pair matching for Charleston Co-morbidity Index (CCI).

AOR, adjusted odds ratio.

^1^Level 1: individual variables as lower level in HLM model

^2^ Level 2: group variables as higher level clusters.

### Seasonal effects on hip fractures and their short-term and long-term morbidity

**Fig 3:**
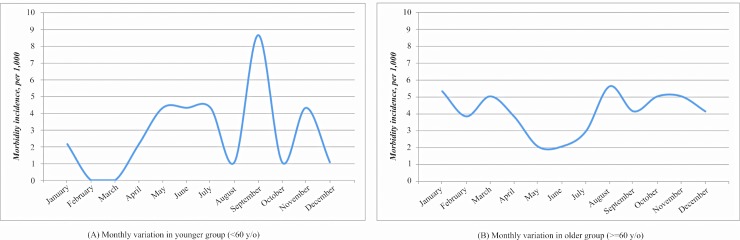
Seasonal effects on hip fractures differentiated by time period and by age group.

The incidence of subsequent short-term (one month after hip fractures surgery) and long-term morbidity (one year after hip fractures surgery) stratified by follow-up period is shown in Table 3. Seasonal effects on hip fractures and different types of subsequent morbidity differentiated by time period and by age groups are shown in Fig 3A and 3B. Short-term morbidity was identified within one month of follow-up for treatment of a hip fracture and long-term morbidity was identified within one year of follow-up in the NHIRD system.

**Table 3:** The incidence of subsequent short-term and long-term morbidity stratified by follow-up period.

	Period I: 2000–2004 (latest follow until 2005)		Period II: 2005–2009 (latest follow until 2010)	
	Short-term follow-up	Long-term follow-up	Short-term follow-up	Long-term follow-up
	*Inc*., (95% CI)	*Inc*., (95% CI)	*Inc*., (95% CI)	*Inc*., (95% CI)
Cerebrovascular accident	0.51 (0.13, 0.89)	4.59 (3.48, 5.70)	1.02 (0.67, 1.38)	7.63 (6.69, 8.58)
(ICD codes:430–438)				
Acute coronary syndrome	0.51 (0.13, 0.89)	3.21 (2.27, 4.14)	0.73 (0.42, 1.03)	6.01 (5.17, 6.86)
(ICD codes:410–414)				
Pneumonia	0.36 (0.05, 0.68)	1.53 (0.88, 2.18)	0.66 (0.37, 0.95)	7.04 (6.13, 7.95)
(ICD codes:480–486)				
UGI bleeding	0.15 (0.00, 0.35)	0.51 (0.13, 0.89)	0.26(0.08, 0.45)	1.55 (1.11, 1.99)
(ICD codes:578.9)				
Peptic Ulcer disease	1.02 (0.49, 1.55)	3.50 (2.53, 4.47)	1.32 (0.91, 1.73)	7.63 (6.69, 8.58)
(ICD codes:531–534)				
Renal Insufficiency	0.51 (0.13, 0.89)	2.11 (1.35, 2.87)	0.56 (0.30, 0.83)	3.20 (2.58, 3.83)
(ICD codes: 580–589)				
New fracture(s) of trunks	0.22 (0.00, 0.47)	0.80 (0.33, 1.27)	0.26 (0.08, 0.45)	1.52 (1.08, 1.96)
(ICD codes:805–809)				
New fracture(s) of upper limbs	0.29 (0.01, 0.58)	1.17 (0.60, 1.73)	0.30 (0.10, 0.49)	1.09 (0.72, 1.46)
(ICD codes:810–819)				
New fracture(s) of lower limbs	1.75 (1.06, 2.44)	5.03 (3.87, 6.19)	1.16 (0.78, 1.54)	4.59 (3.85, 5.34)
(ICD codes: 820–829)				

***Inc***., incidence; 95% ***CI***: confidence intervals

To investigate the incidence of subsequent short-term and long-term morbidity, the period of the study was further divided into two sub-periods, 2000–2004 (period I) and 2005 to 2009 (period II). In long-term follow-up, the incidence of cerebrovascular accident (CVA) was ranked first in both sub-periods ds (4.59% for period I; 7.63% for period II). Among nine major categories of complicated morbidity associated with hip fractures, the incidence of five long-term morbidities including CVA, acute coronary syndrome (ACS), pneumonia, peptic ulcer and UGI bleeding was significantly higher in period II (2005–2009) than period I (2000–2004) based on the comparison of their 95% CIs. The most common subsequent short-term diagnoses were new fractures of the lower limbs (cumulative incidence 1.75%, 95% CI: 1.06–2.44%), peptic ulcer disease (1.02%, 0.49–1.55%), and other chronic comorbidities including CVA, ACS, and renal insufficiency (0.51%, 0.13–0.89%). The distribution of subsequent short-term morbidity was consistent in both sub-periods.

The seasonal variation in short-term complications in younger and older adults is shown by age stratum in Fig 3A and 3B. The incidents in first and fourth seasons are slightly higher in older age group by chi-square test (p = .05). The highest numbers of complications occurred between September and January and the lowest between second and third seasons. When stratified by age, the summer-winter variation was significant in the elderly for short-term complications. Younger adults had a higher proportion of new fractures of lower limbs than did the elderly. The most common complications after hip fractures in the elderly were peptic ulcer disease, lower limb fractures, and cerebrovascular accidents.

## Discussion

Hip fractures are a major cause of disability and the most costly type of fracture for the health care services [21], especially for an aging society. The deterioration controls and magnitude of risk management to prevent long-term adverse effects after hip fracture surgery are urgently public concerns. Based on nationwide data, our study documented the year trends of incidence of hip fractures and subsequent morbidity in Taiwan between 2000 and 2010. This national database was established by evidence-based records and doctor-recognized diagnoses which are more reliable and accurate than self-reported diseases in a primary survey. This longitudinal study has several strengths. Large data analysis has shown great potential in underlying valuable insights. Few previous studies were conducted using this method with such a large amount of medical data and providing clear information to their long-term impact to guide clinical practitioners [22]. A complete understanding of prognostic information related to outcomes is required and incorporated in order to reduce the burden of hip fractures and to plan strategies for care.

In northern Europe, the majority of hip fractures occurred in females in the oldest age stratum. Among the elderly, unpredictable falls due tofatigue may cause a fatal fracture, especially in elderly women. The lifetime risk of a hip fracture is 16%-18% in white women and 5%-6% in white men. The result of our study is consistent with a 10-year, nationwide study of community-dwelling elders in Switzerland which indicated that age at the time of hip fracture was gender-specific. This result was also in-line with another secondary data analysis in Norway [11].

According to one recent study, rural people had a higher rate of hospitalization but a lower mortality rate after falls than did non-rural people [23]. The same aforementioned Switzerland study also showed the effect of the area-level income. Those with the highest or medium income had a lower incidence of hip fractures compared with those from the lowest income category [24]. However, in our study, there was no socio-economic difference. This might be related to nearly universal national insurance access and fewer economic barriers in Taiwan.

Hip-fractured patients undergoing surgery might have mobile limitation during a short period after operated treatment. However, if they reject the operation, they may suffer paraplegic in the future and will have more related complications in the long run. According to our study, most trochanteric fractures received the same treatment, i.e. open reduction.

The short-term complications in younger and older adults were shown to have different seasonal variations. The most common subsequent comorbidities were pneumonia and new upper-limb fractures in younger adults. In the elderly, the most common comorbidity was pneumonia, followed by peptic ulcer and cerebrovascular accident. Hip and acetabular fractures are high energy blunt injuries and most of the elderly patients sustaining hip fractures were at high risk for associated thrombosis, which strongly influenced their outcome after fractures.

Over time, the number of subsequent morbidities increases in trauma patients [25]. In the United States, although the age-adjusted incidence of hip fracture and subsequent mortality in persons 65 and older is steadily declining, all comorbidity in patients with hip fractures has increased from 1995 to 2005 [25]. Risk factors should be well known in these specific target epidemics in order to develop interventions to prevent fractures and subsequent complications [26].

Another population-based study in Norway found no differences in the incidence of hip fractures between urban and rural regions [11], but there was a significant seasonal variation in hip fractures occurring in outdoor areas, especially in the winter [11, 19]. Although there was no severe weather in Taiwan, we also found that the incidence of hip fractures and subsequent complications occurred with a seasonal difference. We observed the variance in difference season in both age groups. However, the difference is not statistically significant. This result is not surprised as the season doesn’t vary significantly in Taiwan. However, we do observe that the incident in first and fourth seasons are higher in older age group. This might be explained by that the older people are more sensitive to the cold weather in these two seasons.

In addition, several studies have emphasized the wide geographic variation in the causes of hip fractures [27]. Compared to developing countries, industrialized countries such as those in northern Europe and the US had a higher incidence of hip fractures [8]. The possible causes of this geographic trend are the north-south gradient, latitude, and environmental factors [19, 27].

Furthermore, osteoporosis has been a major problem related to age-related fractures in the population with age over 65 in Taiwan. Most of the age-related fracture were diagnosed and treated without further investigating the underlying cause (osteoporosis) [28]. Therefore, the number of osteoporosis shown in NHI database might be highly underestimated. As a result, this could mislead the interpretation of osteoporosis leading to comorbidities. However, osteoporosis related fractures are a viable topic and will be investigated in the future study [16, 29].

In summary, hip fractures and subsequent morbidity are increasing in Taiwan’s aging society. Older age, female gender, and time periods were independent risk factors for subsequent morbidities after surgery. A summer-winter variation in short-term and long-term morbidity is observed among the elderly.
